# Fibroblasts are not just fibroblasts: clear differences between dermal and pulmonary fibroblasts’ response to fibrotic growth factors

**DOI:** 10.1038/s41598-023-36416-6

**Published:** 2023-06-09

**Authors:** Sofie Falkenløve Madsen, Jannie Marie Bülow Sand, Pernille Juhl, Morten Karsdal, Christian S. Thudium, Anne Sofie Siebuhr, Anne-Christine Bay-Jensen

**Affiliations:** 1 Department of Biomedical Sciences, University of Copenhagen, Copenhagen, Denmark; 2 Immunoscience, Nordic Bioscience, Herlev, Denmark; 3 Hepatic and Pulmonary Research, Nordic Bioscience, Herlev, Denmark

**Keywords:** Cell growth, Cell migration, Mechanisms of disease, Cell biology, Biomarkers, Gene expression analysis

## Abstract

Systemic Sclerosis (SSc) hallmark is skin fibrosis, but up to 80% of the patients have fibrotic involvement in the pulmonary system. Antifibrotic drugs which have failed in a general SSc population have now been approved in patients with SSc-associated interstitial lung disease (ILD). This indicates that the fibrotic progression and regulation of fibroblasts likely depend on local factors specific to the tissue type. This study investigated the difference between dermal and pulmonary fibroblasts in a fibrotic setting, mimicking the extracellular matrix. Primary healthy fibroblasts were grown in a crowded environment and stimulated with TGF-β1 and PDGF-AB. The viability, morphology, migration capacity, extracellular matrix formation, and gene expression were assessed: TGF-β1 only increased the viability in the dermal fibroblasts. PDGF-AB increased the migration capacity of dermal fibroblasts while the pulmonary fibroblasts fully migrated. The morphology of the fibroblasts was different without stimulation. TGF-β1 increased the formation of type III collagen in pulmonary fibroblasts, while PDGF-AB increased it in dermal fibroblasts. The gene expression trend of type VI collagen was the opposite after PDGF-AB stimulation. The fibroblasts exhibit different response profiles to TGF-β1 and PDGF-AB; this suggests that drivers of fibrosis are tissue-dependent, which needs to be considered in drug development.

## Introduction

Fibrosis is the result of excessive deposition of extracellular matrix (ECM) proteins. The excessive deposition can be caused by dysregulation of fibrogenesis and the wound-healing process. The dysregulation leads to constant activation of the fibroblasts, resulting in an overproduction of ECM proteins. Fibrosis often leads to organ malfunction and can be associated with high morbidity and mortality^1^. The fibroblasts’ origin and ability to adapt their ECM production to the surrounding tissue are often highlighted, while the difference in specific ECM production has not been highly investigated^2,3^. Furthermore, the pathogenesis of fibrotic diseases is not fully elucidated, and currently, no treatments stop fibrogenesis; it only slows it down^4^. Thus, there is a need for a better understanding of the pathogenesis, disease progression, and treatment of fibrosis^4^.

One of the unelucidated fibrotic diseases is systemic sclerosis (SSc), which is a multi-organ disease where the diagnosis is based on the thickening of the skin. Skin thickening has also been shown to correlate with organ involvement and increased mortality^4,5^. In SSc, fibrosis spreads throughout the body, and the lungs are the second most affected organ, in addition to the skin. Up to 80% of SSc patients develop lung fibrosis, while 25% have developed severe lung fibrosis or interstitial lung disease (ILD) within 3 years of the initial SSc diagnosis^5,6^. Pulmonary fibrosis is also the most common cause of death in SSc patients^7,8^. The limited understanding of the underlying pathogenesis of both SSc and ILD makes the diseases difficult to treat^4^. Many antifibrotic drugs have failed when examining a general SSc population^9–12^. However, a more narrow focus on the SSc-associated interstitial lung disease (SSc-ILD) subpopulation has resulted in the approval of two drugs specifically for SSc-ILD: Nintedanib and Tocilizumab^13,14^. Nintedanib was initially approved for idiopathic pulmonary fibrosis (IPF) and has been shown to reduce the decline in forced vital capacity (FVC) in both IPF and SSc-ILD^15,16^. In IPF, the decline in FVC was also consistent with slowing disease progression^15^. Tocilizumab was initially used for inflammatory diseases but has been shown to reduce the decline in FVC in SSc-ILD patients. Based on this, Tocilizumab was approved for SSc-ILD in the US in 2021^13^.

Even though the disease pathogenesis is unclear, multiple fibroblast activation factors, such as transforming growth factor beta (TGF-β) and platelet-derived growth factor (PDGF), have been implicated in both SSc and ILD^1,6,17–20^. A hallmark of fibrotic diseases is continuous fibroblast activation and differentiation into myofibroblasts. The activation leads to excessive production of ECM proteins, such as collagens and fibronectin^21^. As the different tissues require different ECM compositions, the fibroblasts adapt their ECM production to suit the needs of the surrounding tissue for it to fit the particular requirements for rigidity and flexibility^2,3^. The behavior of diseased fibroblasts from both SSc and ILD has been characterized and compared to healthy fibroblasts ^22–26^. Diseased fibroblasts migrate faster, express more type I collagen, and contract more than healthy fibroblasts^22–26^. Moreover, healthy fibroblasts stimulated and cultured in a crowded environment in the Scar-in-a-Jar (SiaJ) model have been suggested as a preclinical model^27–32^. In the model, it has been shown that nintedanib can inhibit TGF-β1 induced fibrosis in both healthy dermal and pulmonary fibroblasts^28,29^.

The aim of this study was to use the SiaJ model to compare the effect of fibrotic growth factors on healthy human dermal and pulmonary fibroblasts, to investigate how similar the response was, and if the response would be similar to diseased fibroblasts.

## Results

### Characterization and comparison of fibroblasts

#### Fibroblast viability

**Figure 1:**
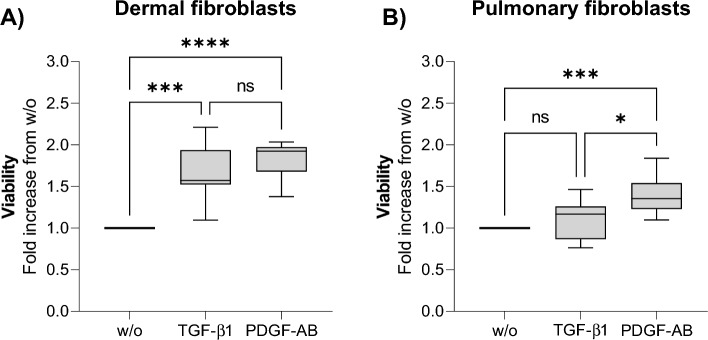
The viability after 12 days of treatment. The viability of dermal (**A**) and pulmonary (**B**) fibroblasts after 12 days of treatment in the SiaJ model. The data are shown as a box plot, with lines indicating the 25-, 50-, and 75-percentiles and the whiskers indicating minimum to maximum of the fold-change to w/o. The data are from three experiments, each with four replicates. The experiments were done on dermal and pulmonary donor one. The data were analyzed by Kruskal–Wallis with Dunn’s multiple comparisons test. *P*-values ≥ 0.05 were not significant (ns). Asterisks indicate: * *p* < 0.05, ** *p* < 0.01, *** *p* < 0.001, **** *p* < 0.0001.

Viability was assessed by the alamarBlue assay on day 12 as a surrogate for metabolic active cells. TGF-β1 increased viability by 1.7-fold in dermal fibroblasts (*p* < 0.001, Fig. 1A). TGF-β1 did not affect the viability of pulmonary fibroblasts compared to w/o (*p* > 0.05, Fig. 1B), but it should be noted that the w/o increases from day 0 to day 12. PDGF-AB increased the viability in dermal fibroblasts by 1.8-fold and by 1.4-fold in pulmonary fibroblasts compared to w/o at day 12 (*p* < 0.0001, *p* < 0.001 respectively; Fig. 1A, B). There was no difference in viability between TGF-β1 and PDGF-AB in dermal fibroblasts. However, in pulmonary fibroblasts PDGF-AB increased the viability more than TGF-β1 (*p* > 0.05 and *p* < 0.05, respectively; Fig. 1A, B).

#### Fibroblast morphology

At the end of the experiment, fibroblast morphology was assessed using Sirius red staining at the bottom of the well. In addition, decellularization was used to assess the ECM deposition more easily.

**Figure 2:**
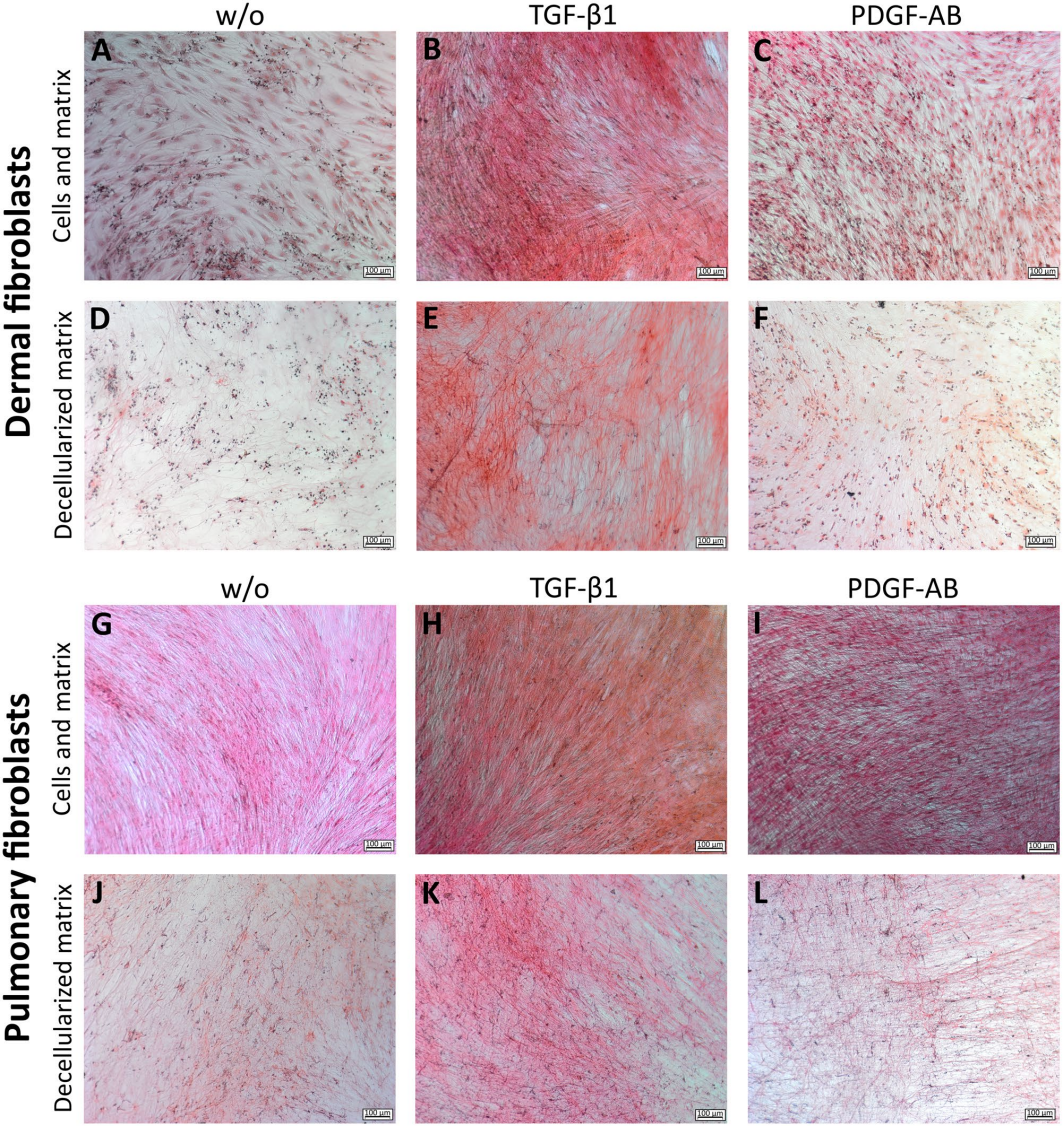
Sirius red staining of fibrillar collagens. Sirius red staining of dermal fibroblasts: untreated (**A**), TGF-β1 treated (**B**) and PDGF-AB treated (**C**). The deceullularized matrix from dermal fibroblasts: untreated (**D**), TGF-β1 treated (**E**) and PDGF-AB treated (**F**). Sirius red staining of pulmonary fibroblasts: untreated (**G**), TGF-β1 treated (**H**) and PDGF-AB treated (**I**). The deceullularized matrix from pulmonary fibroblasts: untreated (**J**), TGF-β1 treated (**K**) and PDGF-AB treated (**L**). The experiments were done on dermal donor one and pulmonary donor two.

After Sirius red staining, the nuclei and outline of the untreated (w/o) dermal fibroblasts were visible (Fig. 2A). The nuclei of the untreated (w/o) pulmonary fibroblasts could also be observed, although the outline of the fibroblasts was not clearly observed (Fig. 2G). The pulmonary fibroblasts were more elongated and narrower than the dermal fibroblasts (Fig. 2A, G). The collagen deposition of the untreated fibroblasts was similar, but the ECM deposition from the dermal fibroblasts did not cover the entire well; the pulmonary fibroblasts’ ECM deposition followed the flow of the fibroblasts and covered the well bottom (Fig. 2D, J).

After Sirius red staining on the TGF-β1 treated fibroblasts, both the dermal and the pulmonary fibroblasts could no longer be differentiated from each other or the matrix, due to changed fibroblast organization and collagen fibers (Fig. 2B, H). The collagen deposition after TGF-β1 treatment was similar between the fibroblast types, leading to the highest amount of fiber deposition compared to both w/o and PDGF-AB (Fig. 2E, K).

After PDGF-AB treatment, the dermal fibroblasts’ outline was more elongated compared to w/o, but both the nuclei and outline of the fibroblasts were visible (Fig. 2C). The number of pulmonary fibroblasts had increased to such an extent that it was difficult to distinguish them from one another after PDGF-AB treatment (Fig. 2I). The collagen deposition after PDGF-AB treatment was higher than in the non-treated fibroblasts but lower than the TGF-β1 induced deposition in both fibroblast types (Fig. 2F, L). 

#### Fibroblast migration

**Figure 3:**
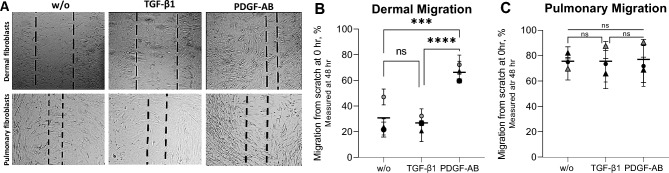
Migration 48 h after the scratch was performed. The scratch after 48 h (**A**). Quantification of the migration of the dermal (**B**) and pulmonary (**C**) fibroblasts, in percent, from scratch at hours 0 to 48. The overall migration mean is shown by a horizontal line. The different experiments are shown by a symbol of the mean ± SD of the four replicates within the experiment. The symbol indicates which donor was used: o = donor one and Δ = donor two. The data were analyzed with Kruskal–Wallis with Dunn’s multiple comparisons test. Not significant (ns) *p* ≥ 0.05. Asterisks indicates: * *p* < 0.05, ** *p* < 0.01, *** *p* < 0.001, **** *p* < 0.0001.

Fibroblast migration was examined in a scratch assay to investigate how the growth factors affect wound closure. The scratch was visually inspected at hours 0 and 48, and the area without fully confluent cells was measured. An example of the visual inspection is shown in Fig. 3.A (see supplementary data for examples of full-size pictures, Figure S5). PDGF-AB increased the migration rate of dermal fibroblasts at 48 h (66%), compared to w/o (31%; *p* < 0.001) and TGF-β1 (27%; *p* < 0.0001, Fig. 3B). However, in the pulmonary fibroblasts, there was no difference in migration between the w/o and treated fibroblasts, as all migrated to the same extent (~ 76%, Fig. 3C).

### Gene expression

The gene expression was quantified in the fibroblasts after treatment with TGF-β1 and PDGF-AB. The targeted genes corresponded to the collagen α-chains and fibronectin, quantified in the biomarkers. The gene expression was quantified after 4 days of treatment to be comparable with the biomarker measurements. For better comprehension, the fold change of gene expression from the control was Log_2_ transformed; thus, a onefold increase in Log_2_ of gene expression translates to a doubling of expression.

**Figure 4:**
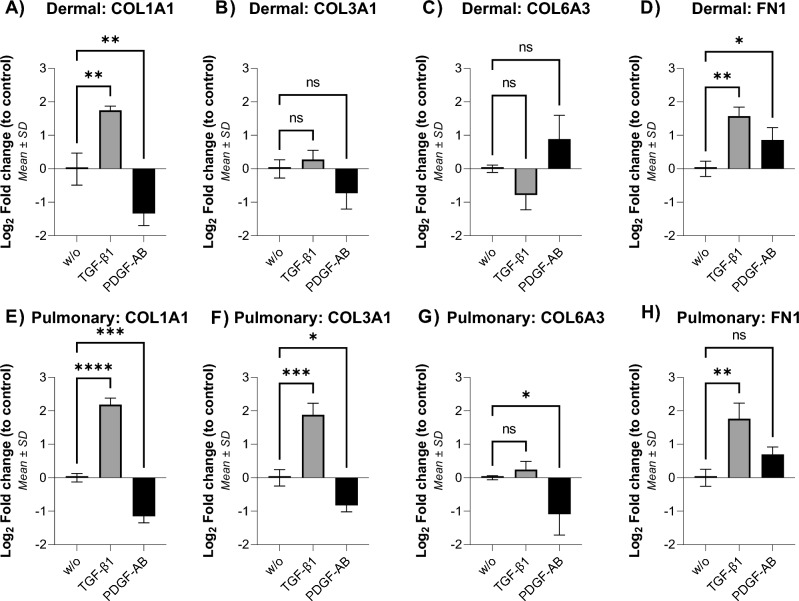
Gene expression of ECM proteins after four days of treatment. Gene expression of type I (COL1A1) (**A**), III (COL3A1) (**B**), and type VI (COL6A3) (**C**) collagen and fibronectin (FN1) (**D**) in dermal fibroblasts. Gene expression of type I (COL1A1) (**E**), III (COL3A1) (**F**), and type VI (COL6A3) (**G**) collagen and fibronectin (FN1) (**H**) in pulmonary fibroblasts. The data are shown as the mean ± SD of triplicates (of triplicates of gene expression). The experiment was done on dermal and pulmonary donor one, and the data were analyzed by one-way ANOVA with Dunnett’s multiple comparisons test against the control. Asterisks indicate: not significant: ns *p* ≥ 0.05, * *p* < 0.05, ** *p* < 0.01, *** *p* < 0.001, **** *p* < 0.0001.

Type I collagen (COL1A1) expression was upregulated by 1.7-fold in response to TGF-β1 in dermal and 2.2-fold in pulmonary fibroblasts (*p* < 0.01 and *p* < 0.0001 respectively, Fig. 4A, E). PDGF-AB led to a 1.3-fold downregulation of COL1A1 in dermal and 1.2-fold in pulmonary fibroblasts (*p* < 0.01 and *p* < 0.001, respectively, Fig. 4A, E). Expression of type III collagen (COL3A1) was unchanged after treatment in dermal fibroblasts, although there was a trend towards downregulation after PDGF-AB treatment (Fig. 4.B). COL3A1 was 1.9-fold upregulated by TGF-β1 and 0.8-fold downregulated by PDGF-AB in pulmonary fibroblasts (*p* < 0.001 and *p* < 0.05, Fig. 4F). Expression of type VI collagen (COL6A3) was unchanged in dermal fibroblasts, although there was a trend of TGF-β1 downregulating and PDGF-AB upregulating COL6A3 (Fig. 4C). In pulmonary fibroblasts, COL6A3 was unchanged after TGF-β1 treatment but 1.1-fold downregulated after PDGF-AB treatment (*p* < 0.05, Fig. 4G). Fibronectin (FN1) expression was upregulated by TGF-β1 treatment by 1.6-fold in dermal and 1.8-fold in pulmonary fibroblasts (*p* < 0.01, Fig. 4D, H). After PDGF treatment, FN1 was upregulated 0.9-fold in dermal fibroblasts but unchanged in pulmonary fibroblasts (*p* < 0.05, Fig. 4D, H).

### ECM turnover

The response of dermal and pulmonary fibroblasts to TGF-β1 and PDGF-AB was investigated in the SiaJ model. The ECM turnover was quantified by measuring the pro-peptides of type I and III collagen (PRO-C1 and PRO-C3, respectively), a signaling molecule released from type VI collagen (PRO-C6, also known as endotrophin), and fibronectin turnover (FBN-C) in the supernatants.

**Figure 5:**
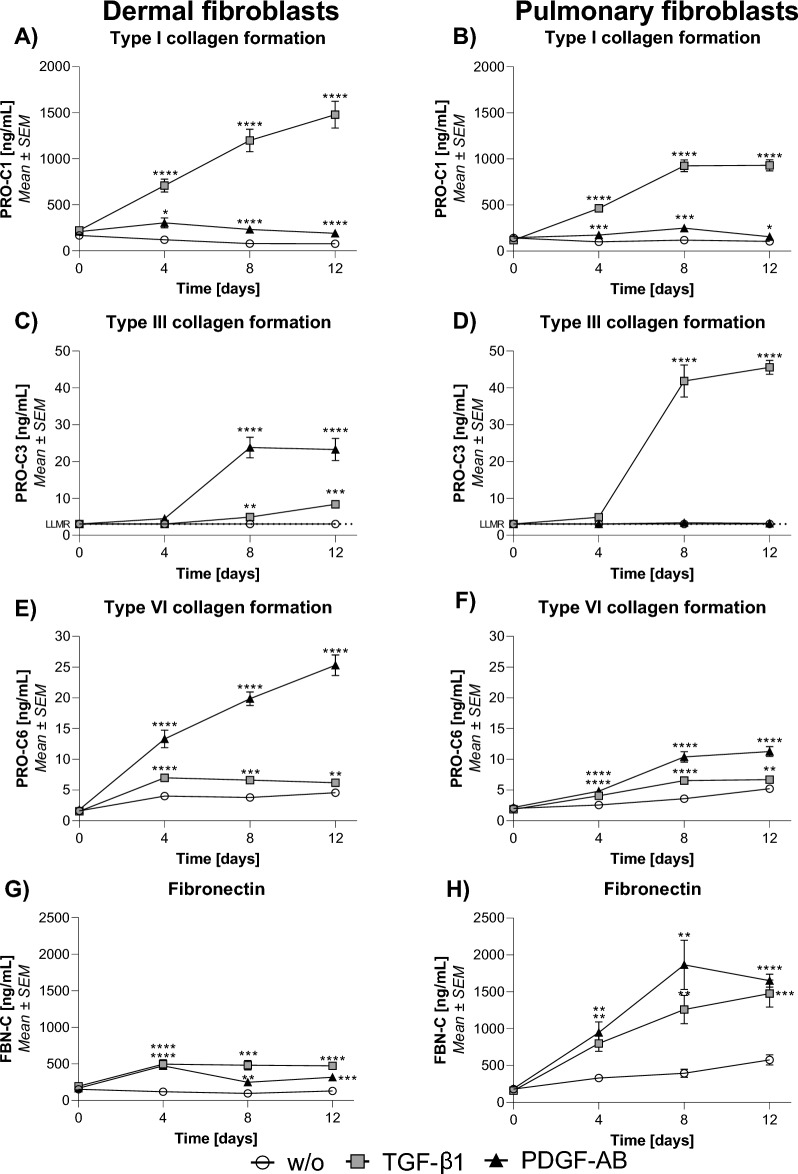
The effect of TGF-β1 and PDGF-AB on collagen formation and fibronectin turnover. Synthesis of type I (**A**), III (**C**), VI (**E**) collagen and fibronectin (**G**) in dermal fibroblasts and type I (**B**), III (**D**), VI (**F**) collagen and fibronectin (**H**) in pulmonary fibroblasts. The data are shown as mean ± SEM of three separate experiments with four replicates each. The experiments are done on dermal and pulmonary donor one. Data were analyzed by two-way ANOVA with Dunnett’s multiple comparisons test against the control. Asterisks indicate: * *p* < 0.05, ** *p* < 0.01, *** *p* < 0.001, **** *p* < 0.0001.

TGF-β1 increased PRO-C1 release at days 4, 8, and 12 compared to the w/o in both dermal (5.8, 15.1, and 19.1-fold, respectively, *p* < 0.0001) and pulmonary (4.6, 7.8, and 8.8-fold, respectively, *p* < 0.0001) fibroblasts (Fig. 5A, B). PDGF-AB increased PRO-C1 on the same days in both dermal (2.5, 2.9, and 2.5-fold, respectively, *p* < 0.05) and pulmonary (1.7, 2.1, and 1.5-fold, respectively, *p* < 0.05) fibroblasts, although to a smaller concentration than TGF-β1 (Fig. 5.A, B). PRO-C3 was increased in dermal fibroblasts on days 8 and 12 after TGF-β1 (1.6 and 2.8-fold, *p* < 0.01) and PDGF-AB treatment (7.8 and 7.6-fold, *p* < 0.0001), thus the latter to a higher concentration (Fig. 5.C). TGF-β1 increased PRO-C3 in pulmonary fibroblasts on days 8 and 12 (13.7 and 14.9-fold, *p* < 0.0001) to a higher concentration than in dermal fibroblasts, while PDGF-AB showed no effect in pulmonary fibroblasts (Fig. 5D). PRO-C6 was increased by TGF-β1 on days 4, 8, and 12 in both dermal (1.7, 1.7, and 1.4-fold, respectively, *p* < 0.01) and pulmonary (1.6, 1.8, and 1.3-fold, respectively, *p* < 0.01) fibroblasts (Fig. 5E, F). PDGF-AB also increased PRO-C6 on the same days but to a higher concentration in both dermal (3.3, 5.3, and 5.5-fold, respectively, *p* < 0.0001) and pulmonary (1.9, 2.9, and 2.2-fold, respectively, *p* < 0.0001) fibroblasts (Fig. 5E, F). FBN-C was increased on days 4, 8, and 12 after TGF-β1 treatment in both dermal (4.1, 5.0, and 3.6-fold, respectively, *p* < 0.001) and pulmonary (2.4, 3.2, and 2.6-fold, respectively, *p* < 0.01) fibroblasts, as well as after PDGF-AB treatment (3.9, 2.6, and 2.4-fold, *p* < 0.01 and 2.9, 4.7, and 2.9-fold, *p* < 0.01, respectively, Fig. 5G, H).

## Discussion

The underlying pathogenesis of both SSc and ILD is currently not understood, but a narrowing focus on SSc-ILD patients has recently led to FDA approval of drugs for the disease^4,13,14^. Biomarkers of type I, III, and VI collagen have previously been reported to be upregulated in both SSc and IPF patients and in dermal and pulmonary fibroblasts^28,29,33–38^. Collagens and fibronectin are a part of tissue homeostasis and an essential part of wound healing^38^. The current study shows that dermal and pulmonary fibroblasts respond differently to the two fibrotic growth factors, TGF-β1 and PDGF-AB, concerning viability, morphology, migratory capacity, gene expression, and production of ECM proteins.

We found that TGF-β1 only increased the viability of dermal fibroblasts, while PDGF-AB increased it in both fibroblast types. Both growth factors were expected to increase viability, which was true for the dermal fibroblasts^28^. Only PDGF-AB increased the viability of the pulmonary fibroblasts, which was also expected from TGF-β1^29^. However, compared to day 0, both w/o and TGF-β1 increased viability. This is only observed in the pulmonary fibroblasts within multiple donors; thus, it cannot be donor variation. The difference between the fibroblast types might be origin specific.

There was a clear difference in morphology between the untreated dermal and pulmonary fibroblasts, as the dermal fibroblasts could be distinguished from each other, while the pulmonary fibroblasts were more elongated and had seemingly grown on top of each other. The color intensity of the collagen deposition, thus, the deposition between the two fibroblast types, is assumingly the same. This might indicate that the pulmonary fibroblasts are more active by default. Neither dermal nor pulmonary fibroblasts differentiate into myofibroblasts without stimulation^28,29^. The amount of collagen deposition was similarly increased in the two fibroblasts by TGF-β1 and PDGF-AB, but the collagen composition might differ. The deposition of the different collagens has previously been investigated, and all showed an increase after growth factor stimulation, while the compositions are difficult to compare^28,29^. Fibroblast migration is an initiating part of the wound healing process, where TGF-β1 and PDGF-AB are released to stimulate ECM production to close the injury site^38,39^. PDGF-AB increased the migration of dermal fibroblasts compared to w/o (66% vs. 31%), which correlates with findings of increased migration by PDGF-BB^40,41^. The low migratory response of dermal fibroblasts to TGF-β1 (27%) stimulation contrasts with the literature^22,41^. TGF-β1 has been shown to increase the migration of healthy and SSc fibroblasts to the same extent^41^. Untreated SSc fibroblasts migrate faster than untreated healthy^22^. Thus, the PDGF-AB stimulated fibroblasts followed the migration pattern of SSc fibroblasts, while the data indicate that the migration capacity of healthy dermal fibroblasts could not be activated by TGF-β1. The pulmonary fibroblasts migrated to the same extent with and without stimulation (~ 76%). The increase by TGF-β1 and PDGF-AB of the migration is consistent with the literature, where TGF-β1 and PDGF-BB have been shown to increase the migration of both healthy and fibrotic pulmonary fibroblasts, the fibrotic to a higher extent^26,40,42–44^. The increase in migration of the untreated pulmonary fibroblasts contradicts the literature, as they usually migrate less than the treated^43,44^. The data indicate that the pulmonary fibroblasts’ migration capacity was already activated or quickly became it. The differing reports on to what extent the fibroblasts are activated suggest that future studies are needed to understand the effect of growth factors on the migration of healthy and diseased fibroblasts. If the migration patterns are the same, using healthy fibroblasts for in-vitro experiments might be preferable, as they are commercially available.

The TGF-β1 stimulated increase of both gene and protein levels of type I collagen and fibronectin in both fibroblast types and of type III collagen in pulmonary fibroblasts correlates with previous findings in SSc, fibrotic pulmonary, and healthy dermal and pulmonary fibroblasts^22–26,28,29,45,46^. The increase of FN1 and FBN-C, PRO-C1 and PRO-C6 in both fibroblast types, and PRO-C3 in dermal fibroblasts by PDGF-AB stimulation is consistent with previous findings^28,41,47^. The downregulation of COL1A1 is contrary to the literature, where PDGF-BB stimulation has been shown to increase type I collagen in dermal and SSc fibroblasts^41^. The downregulation might be because of a high PDGF concentration, as Lepistö et al*.* have shown that low concentrations [1 ng/mL] of PDGF-AB and PDGF-BB upregulate the gene expression of type I and III collagen in wound fibroblasts, while high concentrations [30 ng/mL] downregulate it ^48^. In the present study, a concentration of 3 nM [~ 80 ng/mL] of PDGF-AB was used, which is even higher; thus, the downregulation of the gene expression correlates with the pattern shown by Lepistö et al*.*^48^. The wound healing process may explain the opposite response between PRO-C6 and COL6A3 of the pulmonary fibroblasts^49^*.* Specks et al*.* showed that the gene expression of type VI collagen was upregulated early in wound healing but downregulated later in the process^49^. They also discovered that type VI collagen was upregulated prior to type I collagen, which our results support^49^. We assessed gene expression on day four to compare it with protein formation. However, the differences could also be due to a delay in protein formation in relation to gene expression or that the genes had already been upregulated when assessed on day four^50^.

PRO-C3 production was increased differently in the fibroblasts: pulmonary fibroblasts increased PRO-C3 after TGF-β1 stimulation, whereas dermal fibroblasts increased to the greatest extent after PDGF-AB stimulation. A similar opposing pattern was observed in COL6A3 gene expression, where there was a trend of PDGF-AB upregulating it in dermal fibroblasts while downregulating it in pulmonary fibroblasts. TGF-β1 and PDGF-AB lead to a different ECM response between the fibroblast types, but also with each type. This essentially means that the ECM production varies between the fibroblasts, resulting in a different ECM composition within the different organs. It should also be noted that the dermal fibroblasts have the highest type VI collagen formation, and the pulmonary fibroblasts have the highest fibronectin turnover. Both type VI collagen and fibronectin are important in wound healing: VI collagen is the molecule with the most von Willebrand factor (vWF) binding domains in the body, and the vWF binds to fibronectin to make clots to stop the bleeding after tissue injury^38^.

When translational biomarkers such as PRO-C1, PRO-C3, and PRO-C6 are used in the SiaJ model, it has the potential to be a preclinical model ^28,29^. The increase of PRO-C1 in response to the growth factors correlates with findings of increased PRO-C1 and type I collagen in SSc and IPF fibroblasts compared to untreated healthy fibroblasts^22,24,51^. However, it has also been shown that TGF-β1 stimulates the increase of type I collagen to the same extent in healthy and SSc fibroblasts^23^. However, there are some contradictory data in the patients: Two studies found PRO-C1 to be high in healthy compared to SSc patients, while one study found PRO-C1 to be highest in SSc patients^33,34,52^. In contrast, no difference in PRO-C1 levels has been found between healthy and IPF patients^53^. It has been suggested that type I collagen in serum is derived from bone, which might explain the similarity between healthy and IPF^53^. Additionally, the biomarker of type I collagen degradation, C1M, has been shown to be able to differentiate between healthy, stable, and progressive IPF patients, where the degradation is highest in progressors^37,53,54^. However, only formation biomarkers can be measured in the SiaJ model, as protein degradation is driven by matrix metalloproteinases which originate from cells not present in the model^55^.

In this current study, TGF-β1 and PDGF-AB increased PRO-C6 in both fibroblast types and PRO-C3 in dermal fibroblasts, while PRO-C3 was only increased by TGF-β1 in pulmonary fibroblasts IPF. Both PRO-C3 and PRO-C6 have been shown to be upregulated in SSc and IPF patients compared to healthy patients; additionally, can the levels differentiate between progressors and non-progressors, correlates with skin thickness in SSc and have been associated with mortality in IPF^33–37,52,53^. Both PRO-C3 and PRO-C6 are upregulated in the SiaJ model and in SSc and IPF patients, while the fibrotic profile in SiaJ is growth factor dependent: as both TGF-β1 and PDGF-AB are suspected to be involved in the disease pathogenesis, we speculate that combining the growth factors would give an even more patient-like response. However, more molecules might be needed, as a fibrotic cocktail which additionally contains cytokines, has been shown to induce fibrotic-like changes in precision-cut lung slides from healthy patients^56^. However, the fibrotic response from a single growth factor in SiaJ can still be inhibited by antifibrotic treatment, which correlates with the reduction in biomarkers seen in the treatment of IPF patients^28,29,57^. It is difficult to determine if the *in-vitro* data reflects the processes occurring in patients, as the findings in patients are contradicting. However, we compared the growth factor response of dermal and pulmonary fibroblasts head to head, which contributes to the understanding of how fibroblasts from different origins respond, even though they are in the same settings. This might help us understand the pathogenesis of fibrotic diseases involving both skin and lung, such as SSc-ILD.

The results in this current study showcase the great potential of the SiaJ model to mimic SSc and pulmonary fibrosis *in-vitro*, while there are some limitations: A major limitation was that all experiments were performed in healthy fibroblasts. Additionally, is it not public information exactly where or what layer of skin/lung the fibroblasts were isolated from. Characterizing fibroblasts extracted from patients with SSc, ILD, and SSc-ILD was not possible for the current study. There is a limitation in the interpretation of the collagen deposition in the Sirius red stainings, as it was not possible to quantify the actual collagen deposition or the number of cells.The translation between the gene expression and the protein formation is currently unknown: The gene expression targets non-released proteins, while the biomarkers target peptides released into the supernatant, which are dependent on both protein synthesis and subsequent proper processing. In future studies, it would be interesting to investigate implicated pathways of TGF-β and PDGF, such as the PI3K/AKT and MAPK/ERK pathways.

In conclusion, we have shown that the expression of fibrotic and ECM-associated proteins is dependent on the fibroblast type. This can help us understand the fibrotic pathways and responses within the different organs and suggest that differences in tissue-dependent drivers of fibrosis may explain why fibrotic diseases are difficult to treat. The use of validated biomarkers, which can be used both preclinically and in clinical settings, may aid the translatability of results. We have shown that it is important to investigate fibroblasts of multiple origins, as fibroblasts are not just fibroblasts.

## Materials and methods

### Fibroblast cell culture: Scar-in-a-Jar

Normal healthy human primary dermal fibroblasts (Lonza, Basel, Switzerland, cat. no. CC-2511; one donor and Cell Applications, San Diego, USA, cat. no. 106-05a; one donor) and healthy human primary lung fibroblasts (Lonza, cat. no. CC-2512; two donors) were cultured at a low passage (passage 6–8) (Donor information, see Supplementary Table S1). All donors gave informed consent. The fibroblasts were grown to confluence in 10% fetal bovine serum (FBS) (Sigma-Aldrich, St. Louis, Missouri, USA, cat. no. F7524) in Dulbecco’s modified eagle medium (DMEM) + GlutaMax (Gibco, Life Technologies, Carlsbad, California, USA cat. no. 31966) with 1% penicillin–streptomycin (Sigma-Aldrich, cat. no. P4333). All experimental protocols were approved by Nordic Bioscience A/S ethics committee prior to execution and all methods were carried out in accordance with relevant guidelines and regulations.

The cells were seeded in 48-well plates at 30,000 cells per well in 10% FBS DMEM (high serum medium) and incubated overnight at 37 °C, 5% CO_2_. The next day, the cells were serum-starved in 0.4% FBS DMEM (low serum medium) to avoid serum interference with later measurements. The crowded environment used in the SiaJ model was obtained by adding macromolecules and ascorbic acid to the medium: 0.4% FBS DMEM media with ficoll (ficoll 70, cat. no. 17031050, GE Healthcare, Chicago, Illinois, USA, 56.25 mg/mL; ficoll 400, cat. no. 17030050, GE Healthcare, 37.5 mg/mL) and L-ascorbic acid 2-phosphate (50 µg/mL; Wako, Osaka, Japan cat. no. 013-19,641)^30^. At day 0, 200 μL of the ficoll-enriched DMEM and 100 μL of appropriate treatment media were added to each well. TGF-β1 (R&D Systems, Minneapolis, Minnesota, USA, cat. no. 240-B) and PDGF-AB (R&D Systems, cat. no. 222-AB) were used as fibrotic growth factors. All three isoforms of TGF-β have been tested, and TGF-β1 gave the best response [unpublished data]. All five isoforms of PDGF have been tested in the model, and PDGF-AB gave a higher or equal fibrotic response (Supplementary Figure S1-4). As TGF-β1 and PDGF-AB are the implied isoforms in fibrotic diseases, together with previous investigations, these isoforms were chosen as the fibrotic growth factors^1,28,29,38^. TGF-β1 was used at a concentration of 1 nM in dermal fibroblasts and 0.04 nM in pulmonary fibroblasts, while PDGF-AB was used at a concentration of 3 nM in both fibroblast types. The concentrations targeted the maximum response in relation to biomarker measurements while also aligning with previous investigations and findings in the literature^28,29^ (Supplementary Figure S1-4). Non-treated fibroblasts were used as a control (w/o). Each experiment had four technical replicates of each treatment. The supernatant was collected on days 0, 4, 8, and 12, and new treatments were added. The supernatant was saved at − 20 °C until analysis. The alamarBlue assay was used to assess cell viability. At the end of stimulation, the cells were fixated with 4% formaldehyde (Sigma-Aldrich, cat. no. 100496; hazard risk) for 15 min in a fume hood and stored at 4 °C in Dulbecco’s phosphate-buffered saline (PBS) (Sigma-Aldrich, cat. no. D8537) until staining.

### Scratch assay: migration assessment

The cells were cultured as described above, except the serum starvation was done the same day as the seeding. The treatments were added on day 0, while the scratch was made on day 1: the scratch was made with a tightly held pipette tip through the cell layer. Afterward, the medium was removed, the wells were washed with PBS to remove loosened cells, and then freshly made treatments were added to the wells. The scratches were visually inspected at hours 0, 24, and 48 after the scratch. Pictures were taken with an Olympus DP71 digital camera connected to an Olympus BX60 microscope. Between the visual inspections, the cells were incubated at 37 °C, 5% CO_2_. Afterward, the scratches were quantified with ImageJ (National Institutes of Health^58^), where the scratch distance was measured as the average of three measurements at each time point. The migration was calculated as percent migration from the scratch edges at time point 0.

### Viability

The alamarBlue assay (Invitrogen, Carlsbad, California, USA, cat. no. DAL1100) assessed the cells’ viability at the beginning and end of the experiment. The viability is based on the reduction of resazurin to resorufin by metabolic active cells. The cells were incubated in 10% alamarBlue in 0.4% FBS DMEM for 2 h at 37 °C, 5% CO_2_. The conditioned media were read using 540 nm as the excitation wavelength and 590 nm as the emission wavelength on a fluorescence microplate reader (SpectraMax, Molecular Devices, San Jose, California, USA). If no fluorescence was detected, the cells were presumed dead. The assay was carried out according to the manufacturer’s guidelines.

### Biomarkers: Enzyme-linked immunosorbent assays

In the supernatant from the cell experiment, biomarkers of ECM formation and turnover were measured using technically validated competitive enzyme-linked immunosorbent assays (ELISAs) (Nordic Bioscience, Herlev, Denmark). Type I, III, and VI collagen formation (PRO-C1 cat. no. 2800, PRO-C3 cat. no. 1700, and PRO-C6 cat. no. 4000, respectively) and fibronectin turnover (FBN-C, cat. no. 0101) were investigated^59–62^. All biomarkers were run according to the manufacturer’s instructions. Briefly, the biomarkers were measured using antigen-coated 96-well plates. Appropriate standards, quality controls, and samples were added with the subsequent addition of peroxidase-conjugated monoclonal antibodies. The plates were incubated for 3 h (PRO-C1) or 20 h (PRO-C3, PRO-C6, and FBN-C) at 4 °C. TMB ONE (3,3′,5,5′-tetramethylbenzidine, Kementec, Taastrup, Denmark, cat. no. 4380) was used as the substrate, and 0.18 M sulfuric acid (Sigma-Aldrich, cat. no. 30743) as the stopping buffer. Plates were read using 450 nm with 650 nm as a reference on an absorbance microplate reader (SpectraMax, Molecular Devices)^59–62^. A standard curve was generated for each assay through a four-parametric model, from which the sample concentrations were calculated. Samples above the measurement range were diluted and reanalyzed, while samples below the measurement range were assigned the value of the lower limit of detection (LLMR). Samples below the LLMR might still differ in concentration, but the assay is not sensitive enough to capture the difference.

### Decellularization of matrix

On day 12, the wells were washed with PBS. Heated extraction buffer (PBS with 0.5% Triton X-100 (Sigma-Aldrich, cat. no. X100) and 20 nM ammonium hydroxide (NH_4_OH, Honeywell, Charlotte, North Carolina, USA cat. no. 30501)) was added to the designated wells to remove the cells. The plates were incubated at 37 °C until no intact cells were visualized. PBS was added to the wells to dilute the debris, and the plates were stored overnight at 4 °C. The next day, the diluted debris was removed, and the wells were washed with PBS and PBS containing 1 nM calcium chloride (CaCl_2_, Sigma-Aldrich, cat. no. C1016) and magnesium sulfate (MgSO_4_, Sigma-Aldrich, cat. no. 63138)^63,64^.

### Sirius red

The cells and collagens were stained to assess the morphology and the deposition of ECM. On day 12 of SiaJ, the wells containing cells were stained in Weigert’s working solution (hematoxylin, Sigma-Aldrich, cat. no. H3136; ferric chloride, Sigma-Aldrich, cat. no. 12322; and 37% hydrochloric acid, Merck, cat. no. 100317) for 8 min and washed in tap water. Both the wells with and without cells were stained in Sirius Red (Sirius red F3B, Sigma-Aldrich, cat. no. 365548; picric acid, Sigma-Aldrich, cat. no. P6744-1GA) for 1 h and dehydrated with ethanol. Pictures were taken with an Olympus DP71 digital camera connected to an Olympus BX60 microscope. 

### Gene expression

The cells were cultured, seeded, and treated as described above with an upscaled volume fitted to 6-well plates with 300,000 cells per well in 3 mL. There were three technical replicates of each treatment. The cells were lysed with RNeasy Lysis Buffer (RLT buffer, Qiagen, Hilden, Germany cat. no. 79216) on day four so that gene expression and ECM turnover could be compared on the same day. The lysate was homogenized with QIAshredder (Qiagen, cat. no. 79656), and RNA was purified using the RNeasy Mini kit (Qiagen, cat. no. 74104). Everything was carried out according to the manufacturer’s instructions. The RNA was assessed with a DeNovix DS-11 (DeNovix, Wilmington, Delaware, USA), with the quality check assessed by the absorbance ratio of 260/280 nm and the quantity assessed by the absorbance ratio of 260/230 nm. The RNA was normalized to 350 ng of total RNA per reaction using RNase-free water. cDNA was generated by using the SensiFAST cDNA synthesis kit (Bioline, London, England, cat. no. BIO-65054) according to the manufacturer’s instructions. Gene expression was quantified using TaqMan Fast Advanced Master Mix (Thermofisher, Waltham, Massachusetts, USA, cat. no. 4444557) on a Quantstudio 1 (Thermofisher) according to the manufacturer’s instructions. The gene expression was normalized to the reference gene 18S rRNA (Thermofisher, cat. no. Hs99999901_s1), with a quality check between runs based on the standard deviation (SD) and cycle threshold (Ct) of the reference gene. The genes of interest (GOI) were COL1A1, COL3A1, COL6A3, and FN1 (Thermofisher, cat. no. Hs00164004_m1, Hs00943809_m1, Hs00915125_m1, and Hs01549976_m1, respectively). All samples were run in triplicate, and the quality was checked by assessing the SD. The differences were calculated with the comparative ΔΔCt method for each GOI, with the results expressed as a fold change compared to the control (non-treated fibroblasts). For better comprehension, the fold change was transformed to Log_2_ of the gene expression; thus, a onefold increase in Log_2_ of gene expression translates to a doubling of expression. The full data set is available in the supplementary file.

### Statistics

Biomarker levels are displayed as the mean ± standard error of the mean (SEM) of three individual experiments with four technical replicates each. Longitudinal biomarker levels are plotted as line graphs, comparing the biomarker levels in supernatants from days 0, 4, 8, and 12 to the control on a specific day using two-way ANOVA with Dunnett’s multiple comparisons test. The migration is displayed as the mean of three individual experiments, depicting each experiment as a symbol ± SD. The differences between the treatments were compared with a Kruskal–Wallis test. The viability was normalized to control (w/o) to obtain fold differences and compared using the Kruskal–Wallis test. The gene expression is displayed as the mean of three replicates ± SD of the Log_2_ fold change to control. The expression is log-transformed for better comprehension. Graphical illustrations and statistical tests were performed in GraphPad Prism version 9.4 (GraphPad Software). *P*-values < 0.05 were considered statistically significant, and asterisks indicate: * *p* < 0.05, ** *p* < 0.01, *** *p* < 0.001, **** *p* < 0.0001.

## Supplementary Information

Supplementary Information 1.

Supplementary Information 2.

## Data Availability

The gene expression datasets generated and analyzed in the current study are included in the Supplementary data. The remaining datasets are available from the corresponding author upon reasonable request.
