# Supplementary material for: Fibroblasts are not just fibroblasts: clear differences between dermal and pulmonary fibroblasts’ response to fibrotic growth factors
Source: Sci Rep. 2023 Jun 9;13:9411. doi: 10.1038/s41598-023-36416-6 (PMC10256773; doi:10.1038/s41598-023-36416-6)
Supplement: Supplementary file 2 — Supplementary Information 2. [file 41598_2023_36416_MOESM2_ESM.docx]

## **Donors**

**Table S1:** Overview of fibroblasts used in this study: Donor 1 of both skin and lung was used in all experiments, while donor two was used in addition to donor 1 in the migration assessment.

| **Origin** | **Healthy human skin** | | **Healthy human lung** | |
| --- | --- | --- | --- | --- |
| **Donor** | 1 | 2 | 1 | 2 |
| **Supplier** | Lonza | Cell applications | Lonza | Lonza |
| **Catalog no.** | CC-2511 | 106-05a | CC-2512 | CC-2512 |
| **Lot** | 0000509796 | 2942 | 0000548315 | 0000667543 |
| **Age (years)** | 37 | 31 | 52 | 40 |
| **Sex** | Female | Female | Male | Male |
| **Race** | African American | Caucasian | Caucasian | Caucasian |

## **Growth factor dose response**

The following section shows dose-response curves from TGF-β1 and the different isoforms of PDGF to support the concentration used for the experiments used in this article.

### **TGF-β dose response**

A dose-response of TGF-β1 was done in both dermal and pulmonary fibroblasts to investigate at which doses of TGF-β1 the fibroblasts had a fibrotic response. The isoform TGF-β1 was chosen for this study as it is the most commonly used; additionally has, the other two isoforms, TGF-β2 and TGF-β3, been tested in healthy dermal fibroblasts, where they promoted a similar or lower fibrotic response [unpublished data]. TGF-β1 was tested in a dose range from 0.004-2 nM in a 2-fold dilution, giving ten different doses. The dose-response of PRO-C1 is shown below, as TGF-β1 gives the highest increase in PRO-C1 of the biomarkers (Figure S1.A, B). In the longitudinal overview of the SiaJ response, the doses start separating on day 8. As many of the doses give approximately the same PRO-C1 response, an area under the curve (AUC) was made to give a better overview (Figure S1.C, D). A dose-response can be observed from the w/o to around 0.031-0.063 nM TGF-β1, while the doses from 0.063 nM and above show no dose-dependency or difference in PRO-C1 response (Figure S1.C, D).

The standard TGF-β1 concentration used in dermal fibroblasts is 1 nM, which we show here, is within the maximum response area in PRO-C1. The standard concentration of TGF-β1 in pulmonary fibroblasts is 0.04 nM [1 ng/mL], which is right between the area of the dose-response and the maximum response: while there is a statistical difference between 0.031 and 0.063 nM, there is no difference to the concentrations above 0.063 or to below, at 0.016 nM (Figure S1). Therefore, we continued to use the concentration of 0.04 nM of TGF-β1, as it was close to the maximum response of PRO-C1, and it follows the concentration used in previous studies with pulmonary fibroblasts by Rønnow *et al.* [1].


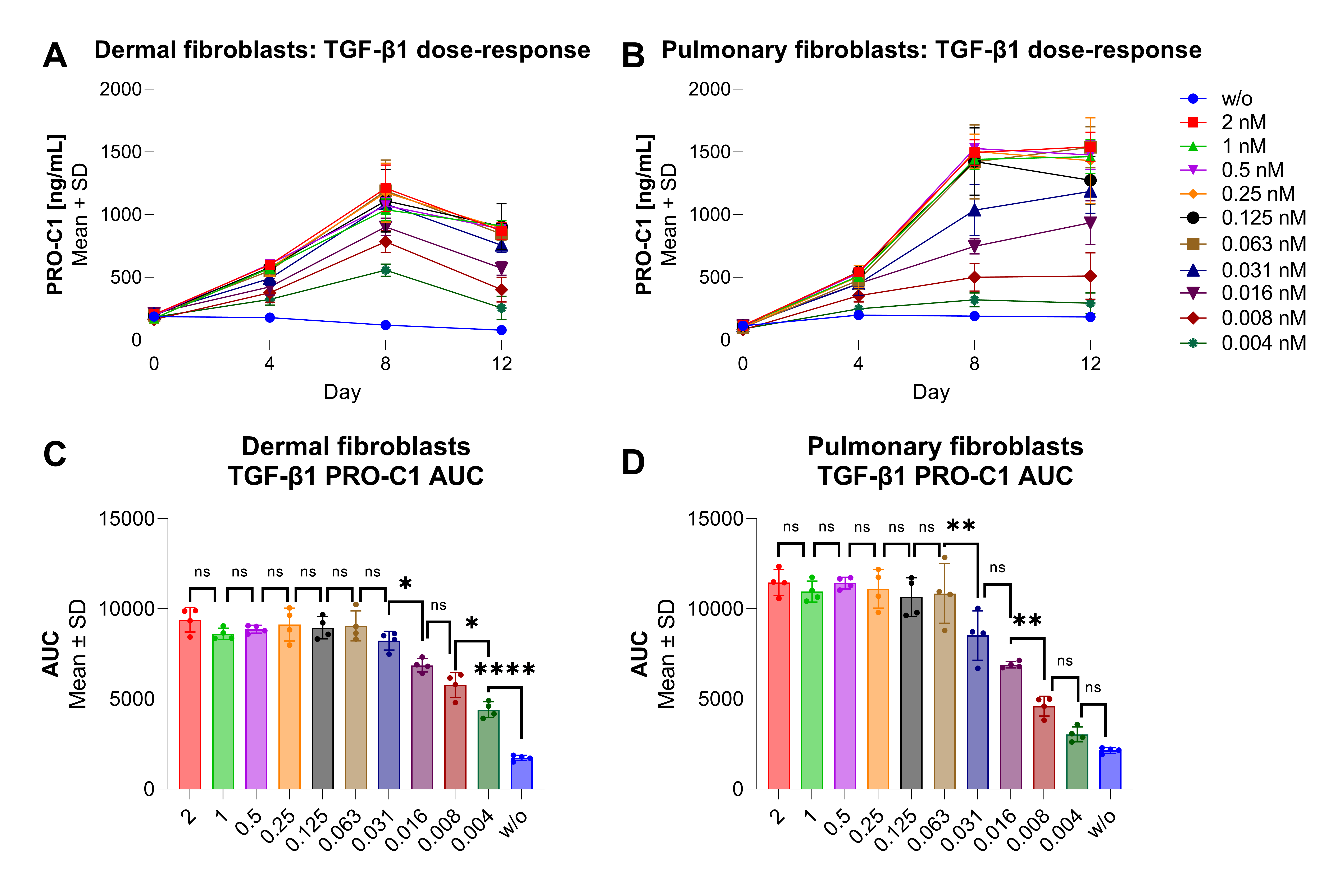


**Figure S1: Dose-response to TGF-b showcased by PRO-C1.** The longitudinal response of dermal (A) and pulmonary (B) fibroblasts to the different doses of TGF-β1. The AUC over the 12 days for dermal (C) and pulmonary (D) fibroblasts. The concentration ranges from 0.004 nM to 2 nM, where the lowest dose is compared to w/o. The experiment was done in donor one in both dermal and pulmonary fibroblasts with four replicates of each concentration. Statistical difference was calculated between the doses beside each other, with one-way ANOVA and Sidak’s multiple comparisons test. Asterisks indicate: not significant: ns p ≥ 0.05, * p < 0.05, ** p < 0.01, *** p < 0.001, **** p < 0.0001.

### **PDGF isoforms dose response**

All the five PDGF isoforms, PDGF-AA, -AB, -BB, -CC, and -DD, were tested in the 3-fold dose range from 0.11 nM to either 3 or 9 nM. The dose-response was done to check the effect of the different isoforms on both dermal and pulmonary fibroblasts.

The most used isoform in the literature is PDGF-BB [2,3]. Here we show that PDGF-AB and PDGF-BB lead to a similar response in the assessed biomarkers. Based on these results as well as previous findings by Juhl *et al.,* we chose to continue with PDGF-AB for further studies [4].

#### **Type I collagen formation, PRO-C1**

As PDGF gives a low response in PRO-C1 compared to TGF-β1, only day 12 was measured when assessing the PDGF doses. The dermal fibroblasts were increased by all five isoforms of PDGF compared to w/o: PDGF-AA to the lowest extent and PDGF-AB to the highest (Figure S2.A). PDGF-AB, -BB, and -CC increased PRO-C1 in pulmonary fibroblasts compared to w/o (Figure S2.B).


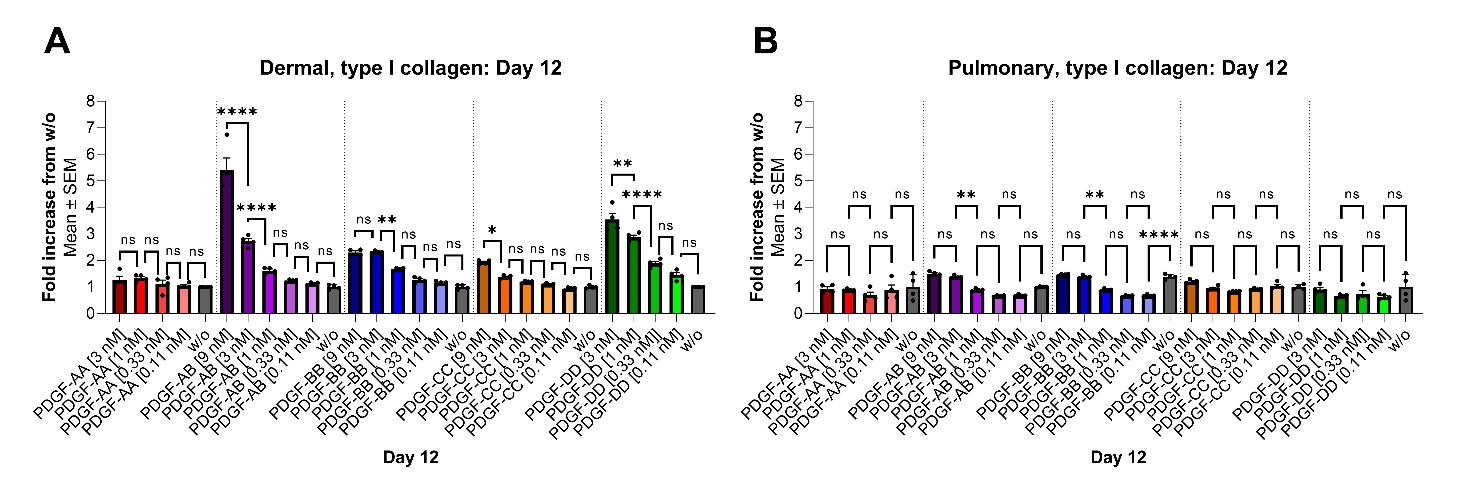


**Figure S2: Dose-response of PRO-C1 to the five PDGF isoforms on day 12.** The response of dermal (A) and pulmonary (B) fibroblasts. The isoforms are separated by a line and colored differently. The experiment was done in donor one in both dermal and pulmonary fibroblasts. The data are shown as mean ± SEM of the fold increase from w/o and dots representing the four replicates of each concentration All data is from measurements on day 12. The dose-response ranges from 0.11 nM to either 3 or 9 nM. Statistical difference was calculated between the doses beside each other, with one-way ANOVA and Sidak’s multiple comparisons test. Asterisks indicate: not significant: ns p ≥ 0.05, * p < 0.05, ** p < 0.01, *** p < 0.001, **** p < 0.0001.

#### **Type III collagen formation, PRO-C3**

The response of PRO-C3 is shown as AUC of the 12 days of the SiaJ model: In the dermal fibroblasts, PDGF-AB, -BB, and -CC increased the PRO-C3 response compared to w/o, while PDGF-AA nor -DD induced it. The highest response of PDGF-AB and -BB was 3 nM and not the highest concentration of 9 nM: this indicates that the maximum response has been met. The highest response of PDGF-CC was 9 nM, while the response of PDGF-CC begins at 3 nM, whereas PDGF-AB and -BB increased PRO-C3 from 1 nM (Figure S3.A). The pulmonary fibroblasts did not respond to any of the PDGF isoforms (Figure S3.B).


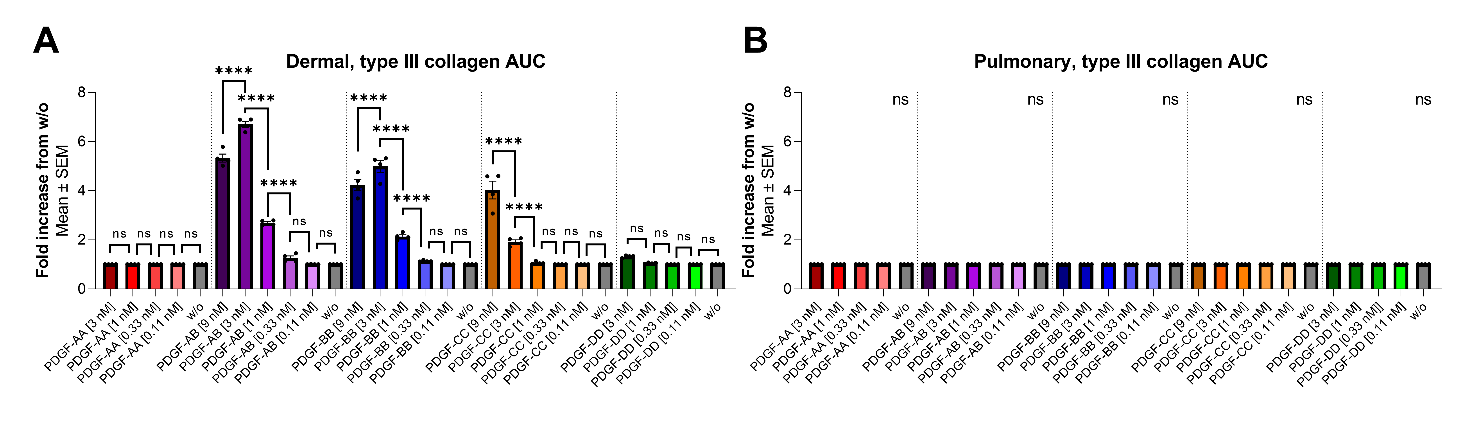


**Figure S3: Dose-response of PRO-C3 to PDGF isoforms.** The response of dermal (A) and pulmonary (B) fibroblasts. The isoforms are separated by a line and colored differently. The experiment was done in donor one in both dermal and pulmonary fibroblasts. The data are shown as mean ± SEM of the fold increase from w/o AUC, with dots representing the four replicates of each concentration. The dose-response ranges from 0.11 nM to either 3 or 9 nM. Statistical difference was calculated between the doses beside each other, with one-way ANOVA and Sidak’s multiple comparisons test. Asterisks indicate: not significant: ns p ≥ 0.05, * p < 0.05, ** p < 0.01, *** p < 0.001, **** p < 0.0001.

#### **Type VI collagen formation, PRO-C6**

The response of PRO-C3 is shown as AUC of the 12 days of the SiaJ model: All isoforms of PDGF increase PRO-C6 in both dermal and pulmonary fibroblasts. PDGF-AA to the lowest extent in both. The dose-response of PDGF-AB and -BB flattened out after 3 nM, while the dose-response of PDGF-CC and -DD still increased at the assessed doses (Figure S4).


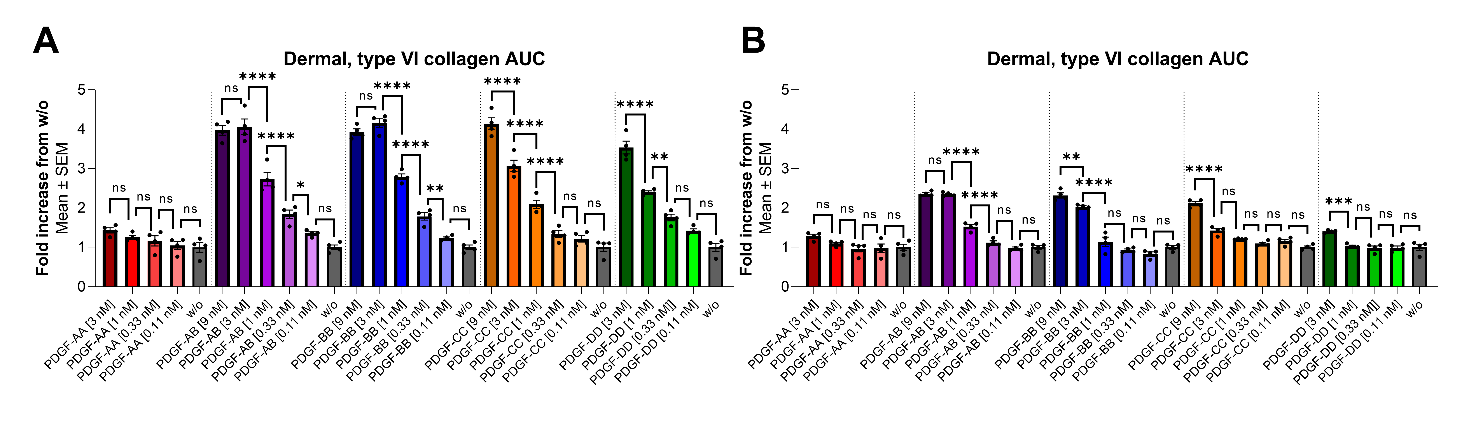


**Figure S4**: **Dose-response of PRO-C6 to PDGF isoforms.** The response of dermal (A) and pulmonary (B) fibroblasts. The isoforms are separated by a line and colored differently. The experiment was done in donor one in both dermal and pulmonary fibroblasts. The data are shown as mean ± SEM of the fold increase from w/o AUC, with dots representing the four replicates within each dose. The dose-response ranges from 0.11 nM to either 3 or 9 nM. Statistical difference was calculated between the doses beside each other, with one-way ANOVA and Sidak’s multiple comparisons test. Asterisks indicate: not significant: ns p ≥ 0.05, * p < 0.05, ** p < 0.01, *** p < 0.001, **** p < 0.0001.

## **Migration**

The pictures in the main article have been cropped, so below is an example of the full-size pictures for the dermal fibroblasts (Figure S5). The dashed lines indicate where the scratch is. The black objects in the pictures are dashed lines which are scratch guidelines drawn under the plates to guide the scratching in the laboratory.

**Figure S5: Full-size pictures of the dermal fibroblasts’ migration over time.** The lines indicate where the scratch was made and how far the cells have migrated. The black areas are the guidelines made prior to the scratch to guide the scratching and to help find the same area at each time point.

## **References:**

[1] Rønnow SR, Dabbagh RQ, Genovese F, Nanthakumar CB, Barrett VJ, Good RB, et al. Prolonged Scar-in-a-Jar: an in vitro screening tool for anti-fibrotic therapies using biomarkers of extracellular matrix synthesis. Respir Res 2020;21:108. https://doi.org/10.1186/s12931-020-01369-1.

[2] Donovan J, Shiwen X, Norman J, Abraham D. Platelet-derived growth factor alpha and beta receptors have overlapping functional activities towards fibroblasts. Fibrogenesis Tissue Repair 2013;6:10. https://doi.org/10.1186/1755-1536-6-10.

[3] Huang J, Beyer C, Palumbo-Zerr K, Zhang Y, Ramming A, Distler A, et al. Nintedanib inhibits fibroblast activation and ameliorates fibrosis in preclinical models of systemic sclerosis. Ann Rheum Dis 2016;75:883–90. https://doi.org/10.1136/annrheumdis-2014-207109.

[4] Juhl P, Bondesen S, Hawkins CL, Karsdal MA, Bay-Jensen A-C, Davies MJ, et al. Dermal fibroblasts have different extracellular matrix profiles induced by TGF-β, PDGF and IL-6 in a model for skin fibrosis. Sci Rep 2020;10:17300. https://doi.org/10.1038/s41598-020-74179-6.
